# Contribution of maternal effects to dietary selection in Mediterranean fruit flies

**DOI:** 10.1111/evo.13664

**Published:** 2019-01-07

**Authors:** Philip T. Leftwich, William J. Nash, Lucy A. Friend, Tracey Chapman

**Affiliations:** ^1^ School of Biological Sciences University of East Anglia Norwich Research Park Norwich NR4 7TJ United Kingdom; ^2^ The Pirbright Institute Woking Surrey GU24 0NF United Kingdom; ^3^ Evolutionary Genomics Group Earlham Institute Norwich Research Park Norwich NR4 7UZ United Kingdom

**Keywords:** Condition dependence, divergence, dietary selection, sexual selection

## Abstract

Individual responses to dietary variation represent a fundamental component of fitness, and nutritional adaptation can occur over just a few generations. Maternal effects can show marked proximate responses to nutrition, but whether they contribute to longer term dietary adaptation is unclear. Here, we tested the hypotheses that maternal effects: (i) contribute to dietary adaptation, (ii) diminish when dietary conditions are constant between generations, (iii) are trait‐specific and (iv) interact with high‐ and low‐quality food. We used experimental evolution regimes in the medfly (*Ceratitis capitata*) to test these predictions by subjecting an outbred laboratory‐adapted population to replicated experimental evolution on either constant high calorie sugar (‘A’) or low‐calorie starch (‘S’) larval diets, with a standard adult diet across both regimes. We measured the contribution of maternal effects by comparing developmental and adult phenotypes of individuals reared on their own diet with those swapped onto the opposite diet for either one or two generations (high and low maternal effect conditions, respectively), both at the start and after 30 generations of selection. Initially, there were strong maternal effects on female body mass and male mating success but not larval survival. Interestingly, the initial maternal effects observed in female body mass and male mating success showed sex‐specific interactions when individuals from high calorie regimes were tested on low calorie diets. However, as populations responded to selection, the effects of maternal provisioning on all traits diminished. The results broadly supported the predictions. They show how the contribution of maternal effects to dietary responses evolves in a context‐dependent manner, with significant variation across different fitness‐related traits. We conclude that maternal effects can evolve during nutritional adaptation and hence may be an important life history trait to measure, rather than to routinely minimize.

Responses of individuals to short‐ or long‐term variation in nutrition are a vital component of host shift biology, the evolution of dietary specialism (Feder et al. 1994; Nosil 2007) and, ultimately, fitness (Slansky Jr. 1982; Raubenheimer et al. 2009; Simpson and Raubenheimer 2012). Nutritional adaptation is frequently key to the successful spread of populations and of population divergence (Schluter 2000; Coyne and Orr 2004; Rundle and Nosil 2005). Understanding nutritional responses and dietary adaptation is also of relevance to improving the control of pest insects (Diamantidis et al. 2011; Panizzi and Parra 2012).

A large body of experimental work has documented the proximate responses of individuals to diet (House 1962; Coudron et al. 2006; Boggs 2009). This has been important in defining the specific ratios of dietary components such as proteins and carbohydrates that maximize the expression of different life‐history traits (Tu and Tatar 2003; Boggs and Freeman 2005; Zajitschek et al. 2009; Joy et al. 2010; Merli et al. 2018). The availability of key nutrients during early life determines characteristics such as developmental speed and survival (Nijhout 2003a) and this has knock‐on impacts on adult traits such as body size (Nijhout 2003b). There is also an extensive literature on the importance of both larval and adult diet on the sexual success of holometabolous insects (and beyond) within the context of condition dependence (e.g., Cotton et al. 2004; Bonduriansky et al. 2015).

It is also well known that traits such as body size and fecundity can be significantly influenced by parental (usually maternal) condition (Mousseau and Dingle 1991; Fox et al. 1997; Davidowitz et al. 2005). Parental effects of age on offspring life history have also been observed (Lind et al. 2015). Maternal effects may be achieved by a variety of mechanisms. A major route is via effects on embryonic growth via maternal modulation of egg cytoplasm composition (Mousseau and Dingle 1991) or by maternally determined differential deposition of mRNAs that direct early embryogenesis (O'Farrell et al. 2004). Paternal effects, mediated by the transit of paternal mRNAs are also possible (Loppin et al. 2005). In this manner, the conditions an individual experiences at the earliest stages of development may be determined largely by the environment experienced by their parents. This will have significant consequences for the proximate responses of offspring to dietary variation.

Despite the huge body of research into the proximate effects of nutrition, we have few data on the evolution of nutritional adaptation, even though it can potentially occur very rapidly, within a few tens of generations (Leftwich et al. 2017). We have even less information on the contribution of maternal effects to such responses. Mostly this is because the contribution of parental effects to the measurement of offspring traits is often intentionally minimized in experimental studies, to maximize the capture of evolved responses and avoid confounding effects on estimates of heritability (Wolf and Wade 2009). This is often achieved through the use of a common garden for raising parental populations, or by rearing test populations through at least two generations on standardized diets. Therefore, an important omission from existing studies is the process of adaptation to novel diets and the role of maternal provisioning in buffering switches between high and low quality nutrient diets.

Strong maternal effects can be selected for where the costs of provisioning by parents are less than those of offspring provisioning, or where parental, but not offspring, environments are resource rich (Mousseau and Dingle 1991; Wolf and Wade 2009; Newcombe et al. 2015). Hence, in situations in which diets remain constant across generations, we would expect selection on maternal effects to be minimized. Any benefits of maternal effects on the expression of resource‐limited life‐history traits in offspring will thus depend upon the extent to which variation in diet quality is shared, or not, across generations. On the other hand, the benefits of maternal effects will be determined primarily by the ratio of costs and benefits in parents versus offspring and if that ratio should change as selection proceeds, even under constant dietary conditions, then maternal effects should evolve (Mousseau and Fox 1998). Maternal effects should be particularly beneficial when there is variation in the nutritional environments experienced by parents versus offspring, particularly so when the nutritional environment of parents, but not necessarily their offspring, is good. The benefits of maternal effects are also expected to depend upon the relative contribution of the traits affected in the offspring to fitness as well as by any trade‐offs with other traits (Gibbs et al. 2010; Khokhlova et al. 2014). This predicts that maternal effects will be trait specific.

To our knowledge, the extent of maternal effect contribution to dietary adaptation has not previously been measured, and this is the omission we tackle in this study. We used the medfly, a notorious generalist and global pest, as our test system. Due to its status as a global pest, there is a wealth of knowledge of the proximate effects of nutrition on key life‐history traits. For example, the medfly exhibits great plasticity in its host selection, utilization (Levinson et al. 1990; Yuval and Hendrichs 2000) and oviposition behavior (Prokopy et al. 1984; Yuval and Hendrichs 2000). Larvae are viable in a wide range of fruits (over 350), from both inside and outside of the known host range (Carey 1984; Krainacker et al. 1987; Liquido et al. 1990). As such, the medfly is of huge economic importance and is an immensely damaging and invasive agricultural pest. Mass rearing strategies have been used extensively as part of sterile insect technique (SIT) control programmes (Robinson 1989). These have yielded extensive data on the importance of larval diet in determining adult body size and mating success and have demonstrated that plasticity in dietary selection and utilization is maintained in domesticated strains (Krainacker et al. 1987; Zucoloto 1993; Nash and Chapman 2014). Importantly, previous research has also documented rapid divergence and local adaptation in response to nutrition, in the same lines as used in this study (Leftwich et al. 2017).

We used an experimental evolution approach, which allowed us to follow in detail the initial stages of adaptation to divergent larval dietary regimes and to measure the ongoing contribution of maternal effects over time. We built upon a recent study that documented divergence and local adaptation in life‐history traits in medfly (Leftwich et al. 2017), using the same experimental evolution lines. We chose to measure features of developmental time and survival as direct measures of response to nutritional quality (Nijhout 2003a), at the initiation of selection and at generation 30, as well as adult traits predicted to be influenced by maternal effects, such as body size and male mating success (Nijhout 2003b). The diets were chosen on the basis that they are known to be able to successfully support larval development, while simultaneously providing qualitative (i.e., carbohydrate form) and quantitative (high vs low calorie) nutritional variation (Nash and Chapman 2014). The ASG, “A” diet is a high calorie mix of simple carbohydrates, while Starch, “S” comprises a lower calorie, complex carbohydrate diet (Leftwich et al. 2017). To document the contribution of maternal effects to each of these traits, we conducted reciprocal diet switches between the A and S diets and measured individuals whose parents were raised on their own regime diets or on the opposite diet for 1 or 2 generations prior to testing (high vs low maternal effect treatments, respectively; Fig. 1).

**Figure 1:**
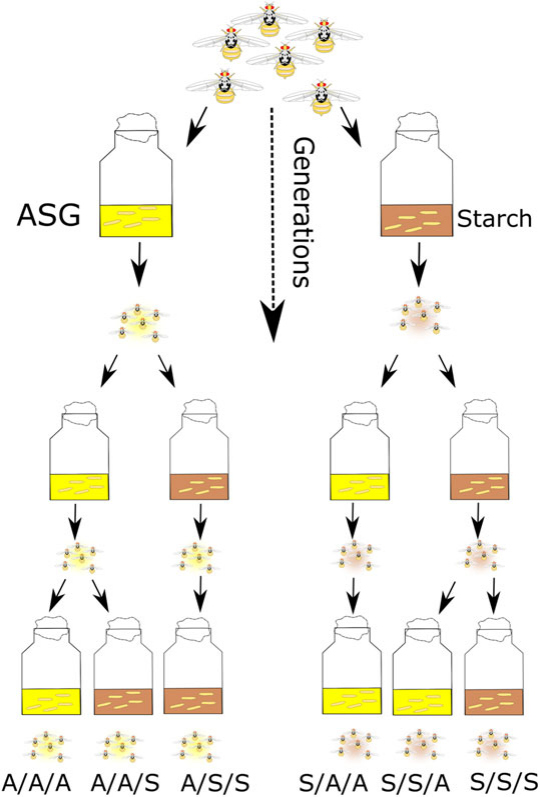
Summary scheme of procedure for measuring the contribution of maternal effects to the evolutionary larval diet manipulations. Two regimes (ASG (A) and Starch (S)) were created from the Toliman wild type, with three independent replicates maintained for each. At early and later generations of experimental evolution, the response of each population was tested on its own medium (A/A/A or S/S/S) or after one (A/A/S or S/S/A; high maternal effect) or two (A/S/S or S/A/A; low maternal effect) generations of rearing on the opposite diet, as indicated.

Specifically, we tested the predictions that maternal effects: (i) contribute to dietary adaptation, (ii) diminish when dietary conditions are constant between generations, (iii) are trait specific and (iv) interact with high‐ and low‐quality food. Overall, the results of our study were largely consistent with these predictions and revealed that the contribution of maternal effects to dietary responses evolved over time, and that maternal effects were trait specific, showed significant context dependence and sex specific variation.

## Methods

We tested the responses of replicated sets of experimental evolution lines adapting to differing larval dietary regimes at the start of selection (generations 3–5 for life‐history traits; 5–7 for mating behavior) and after generation 30. Testing took place after 1 or 2 generations of rearing on the diet opposite to that of the adaptation treatment (high and low maternal effect treatments, respectively; Fig. 1). The low maternal effect data on the effects of the two‐generation food swaps on larval development time, survival, and body mass are taken from a previous study (Leftwich et al. 2017). These already published data are incorporated here into this study to provide a direct comparison with the one‐generation food swap (high maternal effect) treatments that were conducted simultaneously. All other data are presented here for the first time.

### ORIGIN AND MAINTENANCE OF FLY STOCKS

The Toliman wild type (wt) strain, originating from Guatemala and reared in the laboratory since 1990 (Morrison et al. 2009) was used to initiate the experimental evolution lines. For at least two years prior to the start of these experiments the strain was reared on a wheat bran larval diet (24% wheat bran, 16% sugar, 8% yeast, 0.6% citric acid, 0.5% sodium benzoate), with adults fed a 3:1 sucrose: yeast hydrolysate mix. Larval and adult densities were not precisely controlled during the rearing of the Toliman strain, maintaining variation in adult versus offspring diet quality/quantity across generations. To initiate the experimental evolution, flies were established on the following two diets: (i) sucrose‐based “ASG” (A) (1% agar, 7.4% sugar, 6.7% maize, 4.75% yeast, 2.5% Nipagin (10% in ethanol), 0.2% propionic acid) or (ii) “Starch” (S) medium (1.5% agar, 3% starch, 5% yeast, 0.5% propionic acid). Adults from both regimes were again maintained on a 3:1 sucrose: yeast hydrolysate mix throughout rearing. The caloric value of ASG and Starch diets were 684 kcal/L and 291 kcal/L, respectively, estimated from published sources (Leftwich et al. 2017). The rationale for not including a base line nutritional treatment formed around the wheat bran diet was that our main aim was to measure how the magnitude of maternal effects changed over time in high and low calories diets under otherwise controlled conditions rather than to document the relative trajectory of evolution against a third, different diet type.

Three independent biological replicates of each of the two evolutionary regimes were maintained under strict allopatric conditions. All experiments and culturing was conducted at 25°C, 50% relative humidity, on a 12:12 light dark photoperiod. Adults emerging from each replicate were maintained in groups of approximately 30 males and 30 females in 0.8 L plastic cages (11 cm x 11 cm x 10 cm). Adults from all lines received the same standard adult diet (ad libitum access to 3:1 w/w sugar:yeast hydrolysate). Each generation, approximately 500 eggs were placed on 100 mL of the appropriate diet in a glass bottle. When 3rd instar larvae started to “jump” from the larval medium, the bottles were laid on sand and pupae allowed to emerge for 7 days. Pupae were then sieved from the sand and held in 9 mm petri dishes until adult eclosion. Unlike for the source Toliman strain, larval, and adult density was standardized in the dietary regimes, minimizing qualitative, and quantitative variation across generations in the experimental evolution regimes.

### EFFECT OF DIET AND HIGH AND LOW MATERNAL EFFECT TREATMENTS ON DEVELOPMENT

#### Regime, parental, and testing diets

Each of the three independent biological replicates for each of the two dietary regimes was tested at the start (3‐5 generations) and after 30 generations of selection, respectively. Flies were tested following rearing on their own regime larval diet, and by crossing onto the opposite larval diets for one or two generations (Fig. 1), in order to measure the contribution of variation in maternal effects to the overall responses observed. Hence the 6 treatments of flies for each trait comprised regime flies tested on their own regime larval diet (AAA or SSS, simplified to “A” or “S”), regime flies tested following one generation of swapping to the opposite larval diet (regime/parental/testing diet; A/A/S or S/S/A) or regime flies following two generations of swapping to the opposite larval diet (regime/parental/testing diet; A/S/S or S/A/A). A/A/S and S/S/A treatments represented “high” maternal effects, as there could be maternal carry over direct from regime through to testing diet. A/S/S and S/A/A treatments represented ‘low’ maternal effects as maternal carry over effects was minimised by rearing for two generations on the test diet before testing occurred (Fig. 1). At the initiation of selection, we conducted the “on diet” regime tests (“A” and “S” treatments) at generation 3, high maternal effect tests at generation 4 (A/A/S and S/S/A treatments) and low maternal effect tests at generation 5 (A/S/S and S/S/A treatments). At generation 30, all six treatments were tested simultaneously. Adults from all treatments were fed as adults on a 3:1 sucrose: yeast hydrolysate mix.

#### Egg to adult survival, development time, and body mass

Eggs were collected over a 24 h period and counted under a dissecting microscope. These egg samples were then incubated on wet Whatman filter paper (Fisher Scientific) and sealed within ten Petri dishes each containing 40 g of larval food medium (ASG or Starch, 0.2 g/egg, 200 eggs per Petri dish). When third instar larvae started to “jump” from the larval medium, the plates were unsealed and laid on sand, and pupae allowed to emerge for seven days. Each plate was checked daily and the number of new pupae formed was recorded. Pupae from each line were kept and monitored for eclosion, and adults were checked for sex before recording the day of eclosion. Non‐eclosed or partially eclosed pupae casings were counted and then discarded. Development time was recorded as the median time (in days) from egg collection to pupation and adult eclosion for each Petri dish. To measure the effect of larval diet and experimental adaptation on body mass, the dry weights of males and females from the development tests were taken by freezing individuals post‐eclosion at –20°C for 24 h, followed by desiccation at 25°C for 24 h and weighing samples of 100 flies from each replicate/treatment on a BDH DE‐100A micro‐balance.

### EFFECT OF DIET AND HIGH AND LOW MATERNAL EFFECT TREATMENTS ON MATING PREFERENCES

Each of the three independent biological replicates for each of the two dietary regimes were also tested for mating preferences at the start (generations 5–7) and after 30 generations of selection. Flies were sorted by sex within 24 hours of eclosion to ensure virginity. Experimental flies were reared in standard 0.8 L rearing cages as above. One male and one female from each population in each mating test were marked with a spot of red paint on the dorsal side of the thorax, while anaesthetized on ice. Marking of treatments was alternated in order to control for any effects of marking or chilling.

#### Assortative mating tests

In order to test for assortative mating by diet, a quartet mating test design was used. For each test, four 5–7 day‐old males and females were placed together in a mating chamber. The quartets were composed of either four flies reared on their own dietary regimes, or of a male and female reared in their own dietary regime together with a male and female reared for one or two generations on the opposite larval diet. This allowed the effect of adaptation to each diet to be assessed individually, by testing preference of flies reared in regime to both combinations of regime crossing. This created nine treatments of mating quartets (regime diet/parental diet/testing diet | competitor diet): A|S, A/A/S|S, A/A/S|A, A/S/S|S, A/S/S|A, S/S/A|A, S/S/A|S, S/A/A|A, S/A/A|S). Two females were aspirated into each 250 mL transparent plastic mating arena at lights on (09:00). Males were aspirated in 30 minutes later. The identity of the first male and female to mate was recorded along with time of male introduction. Cages were monitored for 3.5 h or when the first mating pair ceased copulation, whichever occurred first. In the generation 5 on diet tests, 60 replicates of the quartet mating tests were conducted per line replicate combination, in generation 6 high maternal effect tests there were 60 replicates each and in generation 7 low maternal effect tests, 20 replicates. In the generation 30 tests (all treatments simultaneously tested), 70 quartet replicates were conducted for flies reared on their own diet and 15 replicates for flies subjected to one and two generation diet switches.

### STATISTICAL ANALYSIS

#### Developmental survival and development time

Developmental survival was measured as proportion data (i.e., the number of individuals that entered a development stage in comparison to those that completed it) and analyzed by generalized linear‐mixed models (GLMMs) using a binomial distribution with the “glmer” function within “lme4” (Bates et al. 2015). Observation‐level random effects were employed to account for overdispersion (identified by comparison of the residual deviance with the residual degrees of freedom). Models with convergence errors were fitted with the “bobyqa” optimizer. Development time (median days to the nearest 12 h) was analyzed by using linear‐mixed models (LMM). Body mass (dry weight) was analyzed by LMM. For an overview, the data from early and late generations were first analyzed together with generations, regime, parental, and proximate test larval diet as fixed effects, with replicate lines nested as a random effect within larval diet selection regime. To increase resolution, the dataset was then split and the dietary responses of flies at early versus late generations analyzed separately. Model selection was conducted by sequential likelihood ratio testing using lmertest: anova (Kuznetsova et al. 2017). After each model of developmental data was fitted, a marginal *r^2^* value was calculated to express the variance explained by the fixed factors using “MuMIn” (Barton 2016). Significance of treatment comparisons was assessed using Tukey HSD multiple comparison tests using the “glht” function within “multcomp” v1.4‐7 (Hothorn et al. 2008). All developmental data analyses were conducted in R v3.3.3 (R Core Team 2017).

#### Mating tests

The number of observed and total possible pairings for each pair type was calculated for each replicate. These raw data were then analyzed using JMATING v1.0 (Carvajal‐Rodriguez and Rolan‐Alvarez 2006). This allows the calculation of coefficients based on modifications to a standard cross product estimator of isolation (Rolán‐Alvarez and Caballero 2000): the pair sexual isolation index (PSI), sexual selection index (PSS), and total isolation index (PTI). PSI is the number of observed matings for each pair type divided by the expected number of matings within these mating pairs. Assuming random mating, it measures sexual isolation. PSS is the expected number of mating pairs within the observed mating frequencies divided by the expected number of pair types from the total potential mates. Under the assumption of random mating, PSS measures the effect of sexual selection. PTI is the product of PSI and PSS (PSI × PSS = PTI), and is the number of observed mating pairs for each pair type divided by expected numbers of mating pairs from the total potential mates. It combines the effects of sexual isolation and sexual selection to describe overall isolation (Rolán‐Alvarez and Caballero 2000; Coyne et al. 2005). Nonparametric G tests, also calculated in JMATING, were used to test for deviations from random mating across the whole coefficient dataset for each mating test. The G test is additive, which allows the contributions of sexual isolation and sexual selection to total isolation to be distinguished.

## Results

### CONTRIBUTION OF MATERNAL EFFECTS TO DEVELOPMENT

#### Developmental survival

**Figure 2:**
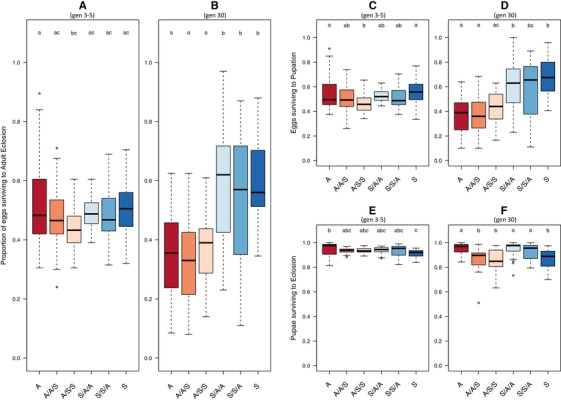
Proportion of Medfly individuals surviving between each developmental stage at generations 3–5 and 30 of artificial selection on divergent larval diets. Boxplots showing developmental survival of individuals derived from A or S larval dietary regimes and maintained on A or S, or crossed to larval diets of either A or S for one (A/A/S, S/S/A) or two (A/S/S, S/A/A) generations. Panels A (3–5 generations) and B (30 generations) show egg to adult survival; Panels C (3–5 generations) and D (30 generations) show larval survival (egg to pupation); Panels E (3–5 generations) and F (30 generations) show pupal survival (pupae to adult). Lower case letter groupings denote significant differences at *P < *0.05 following post hoc analysis with “multcomp.” Outliers are marked when > 1.5 *interquartile range (IQR). The data on the effects of on‐diet and the two‐generation food swaps (low maternal effects) are taken from a previous study (Leftwich et al. 2017), one‐generation food swaps (high maternal effects) are shown for the first time.

*Overall egg to adult survival*: At the start of selection (generations 3–5) there was no evidence for any significant differences due to maternal effects and overall, individuals from the “A” regime reared on a “S” testing diet for two generations had a significantly lower developmental survival (glmer; regime × testing diet; *z = *3.32, *P < *0.001, *r^2^* = 0.004, Table S1A, Fig. 2A). At generation 30, there was also no effect of maternal effect variation (Fig. 2B). Instead the evolutionary regime from which the flies were derived was the only significant predictor of egg to adult survival, with “A” regime derived flies showing a lower survival than all “S” regime flies (glmer; regime; *z* = 8.01, *P* < 0.001, *r^2^* = 0.062, Table S1B, Fig. 2B). Hence, the egg to adult survival of individuals was unaffected by swapping to the opposite diets and instead showed evidence of an evolved dietary difference, with consistently higher survival in “S” over “A” individuals, as previously described (Leftwich et al. 2017). This pattern was largely attributable to larval survival, as described below. *Larval survival*: The results for just larval survival itself (number of eggs that reached pupation) were broadly consistent with overall egg to adult survival (Fig. 2C, D). Again, there was no evidence for high or low maternal effect differences at the start or after 30 generations (Table S1C, 1D, Fig. 2C, D). *Pupal survival*: that is, the number of pupae that became adults, was high across all treatments (Fig. 2E, F). There was a significant effect of proximate test diet at the start (glmer; testing diet; *z* = 4.01, *P < *0.001, *r^2^* = 0.01, Table S1E), which became more pronounced at generation 30 (glmer; testing diet; *z* = –7.87, *P* < 0.001, *r^2^* = 0.075, Table S1F). However, as before, there was no effect of high or low maternal effect treatments and no evidence of dietary adaptation affecting pupal survival over successive generations. Overall flies tested on the A diet had the highest pupal survival regardless of the evolutionary diet regime from which they were derived (Fig. 2E, F).

#### Development time

**Figure 3:**
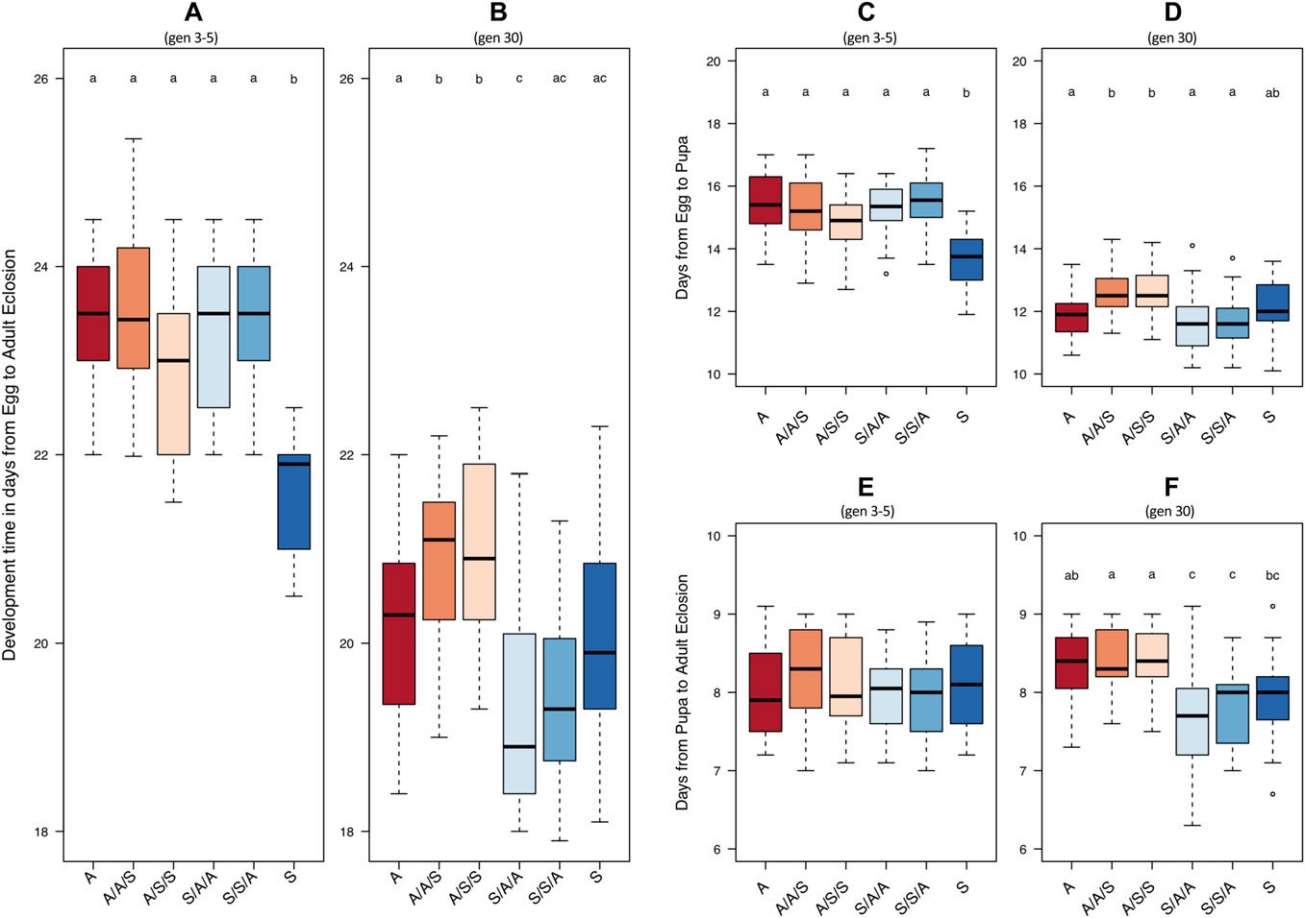
Time (in days) for Medfly individuals to complete each developmental stage at generations 3–5 and 30 of artificial selection on divergent larval diets. Boxplots showing developmental time of individuals derived from A or S larval dietary regimes and maintained on A or S or crossed to larval diets of either A or S for one (A/A/S, S/S/A) or two (A/S/S, S/A/A) generations. Panels A (3–5 generations) and B (30 generations) show egg to adult development time; Panels C (3–5 generations) and D (30 generations) show larval development time (egg to pupation); Panels E (3–5 generations) and F (30 generations) show pupal development time (pupae to adult). Lower case letter groupings denote significant differences at *P* < 0.05 following post hoc analysis with “multcomp.” Outliers are marked when > 1.5 *IQR. The data on the effects of on‐diet and the two‐generation food swaps (low maternal effects) are taken from a previous study (Leftwich et al. 2017), one‐generation food swaps (high maternal effects) are shown for the first time.

*Overall egg to adult development time*: at the start of selection, development time was significantly influenced by an interaction between both evolutionary diet regime and testing diet (lmer; regime × testing diet; *t_180_* = –3.59, *P < *0.001) and a maternal effect arising from an interaction between parental diet and testing diet (parental × diet; *t_180_* = 2.26, *P < *0.02) (*r^2^* = 0.388, Table S2A, Fig. 3A). By generation 30 this maternal effect interaction had disappeared and development time was determined solely by significant main effects of regime and testing diet, indicating a reduction in the extent of maternal effects as evidence for adaptation to the respective diets started to emerge (lmer; regime; *t_162_* = –4.87, *P < *0.001, diet; *t_162_* = 4.72, *P* < 0.001, *r^2^* = *0.3*, Table S2B, Fig. 3B) (Leftwich et al. 2017). This pattern appeared to be attributable to both larval and pupal development, as described below. *Larval development time*: that is, from egg to pupation, was initially significantly influenced by an interaction between both evolutionary diet regime and testing diet, but with no evidence of a parent × diet interaction (Table S2C, Fig. 3C). At the start of selection, “S” regime flies reared on “S” had significantly faster development than any other treatment. However, following selection, an effect of testing diet was the only significant predictor of development time, with individuals reared on “A” developing significantly faster than those reared on S, regardless of evolutionary regime (Table S2D, Fig. 3D) and indicating a potential switch over in developmental speed between the different diets. *Pupal development time*: The time spent in pupal development showed a contrasting pattern. At the start of selection, there was no effect of any treatment on development time (Table S2E, Fig. 3E). However, at generation 30, there was a significant main effect of evolutionary regime diet (lmer; regime; *t_162_* = –6.87, *r^2^* = 0.226, *P < *0.001, Table S2F, Fig. 3F), with “S” having a significantly shorter pupation time than “A” individuals, providing some evidence for divergence in pupal development time across the different diets.

#### Body mass

**Figure 4:**
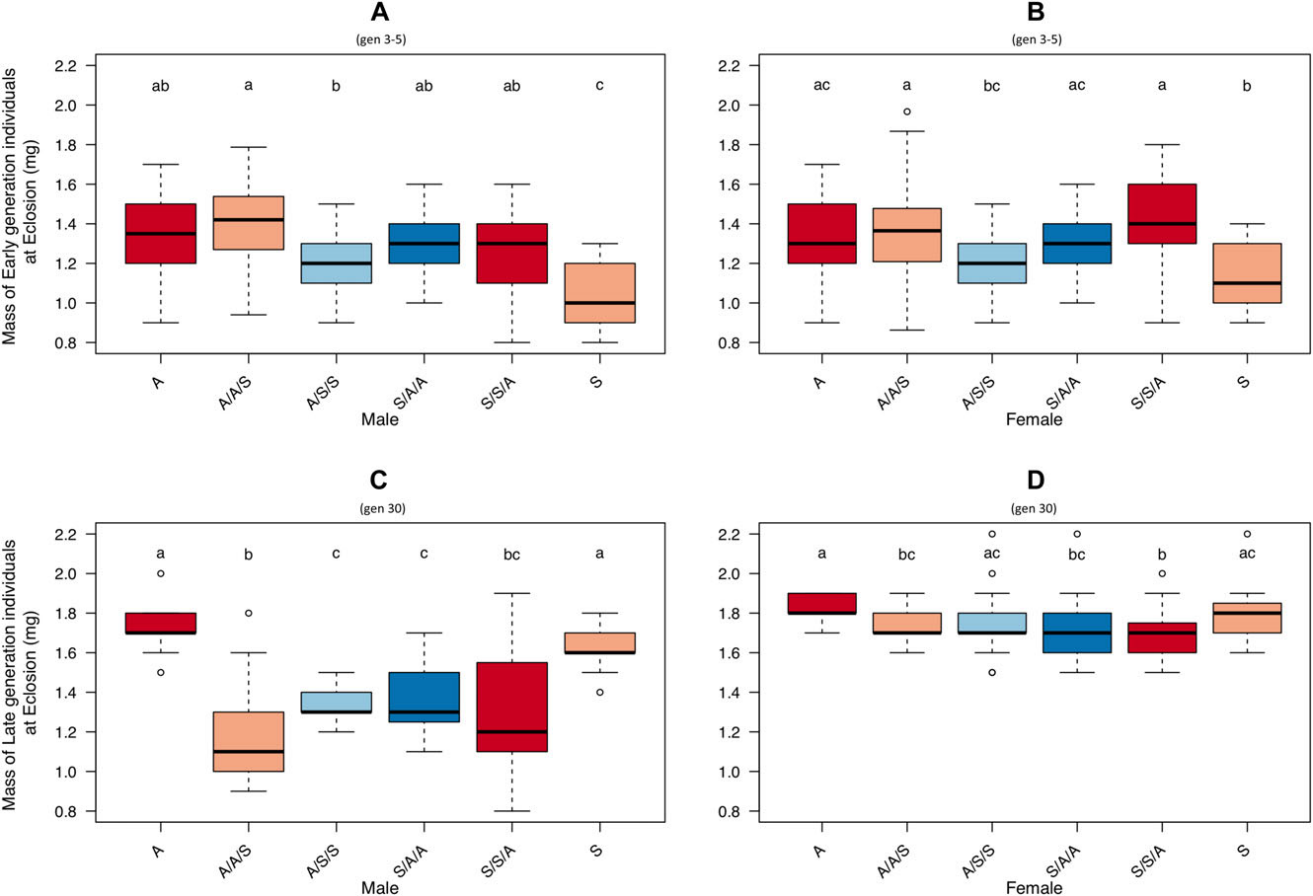
Mass at eclosion of Medfly males and females when at generations 3–5 and 30 of artificial selection on divergent larval diets. Mass at eclosion of individuals derived from A or S larval dietary regimes and maintained on A or S or crossed to test larval diets of either A or S for one (A/A/S, S/S/A) or two (A/S/S, S/A/A) generations. Top row = early generations (3–5), panel A shows mass at eclosion for males, B shows mass at eclosion for females. Bottom row = late (30) generations of selection. Panel C shows mass at eclosion for males, D shows mass at eclosion for females. Lower case letter groupings denote significant differences at *P* < 0.05 following post hoc analysis with “multcomp.” Outliers are marked when > 1.5 *IQR. The data on the effects of on‐diet and the two‐generation food swaps (low maternal effects) are taken from a previous study (Leftwich et al. 2017), one‐generation food swaps (high maternal effects) are shown for the first time.

Body mass was initially determined by evolutionary regime, parental, and testing diet, that is by the strength of maternal effects (Male body mass lmer; regime; *t_174_* = –3, *P* = 0.012, parental; *t_174_* = –3.55, *P < *0.001; testing diet; *t_174_* = –2.71, *P* = 0.007, *r^2^* = 0.248, Table S3A, Fig. 4A: Female body mass lmer; parental × testing diet; *t_180_* = ‐5.05, *P* < 0.001, *r^2^* = 0.191, Table S3B, Fig. 4B). Lines reared on “S” for two successive generations or more (i.e., the A/S/S and “S” treatments) had the lowest body mass, while those reared on “A” the highest. By generation 30, male body mass was influenced by the same dietary factors as at the start, but in the opposite direction (lmer; parental × testing diet; *t_162_* = 3, *P < *0.003; regime x testing diet; *t_162_* = 9.65, *P* < 0.001, *r^2^* = 0.537, Table S3C, Fig. 4C). Female body mass at generation 30 was predicted by an interaction of regime x testing diet (lmer; regime x testing diet; *t_156_* = 4.81, *P* < 0.001, *r^2^* = 0.145, Table S3D, Fig. 4D) with no evidence for any influence of maternal effect variation. Overall, individuals showed a reduction in body mass when reared on the opposite diet and this effect was more prominent in males. This shows the effect previously described for body size adaptation, with individuals being larger when reared on their own type of food (Leftwich et al. 2017). In terms of the contribution of maternal effect variation, at the start of selection, individuals from the “A” regime tested on “S” diets in the high maternal effect treatments retained a heavier body mass that was similar to individuals remaining on the “A” diet. The low maternal effect flies were significantly lighter. There was no such effect in “S” flies tested on “A.” However, by generation 30, the pattern was significantly altered, with a strongly divergent response among the sexes. Males of either regime had a significantly lower body mass when reared “off‐diet” under high or low maternal effects, while females showed little difference in body mass between treatments.

Overall the results were consistent with the predictions, that maternal effects can evolve (i.e., they disappeared by generation 30 in “A” regimes), that their contribution diminishes under conditions where dietary variation is removed and that the contribution of maternal effects is context dependent and influenced by qualitative and quantitative variation in diets (i.e., occurred in “A” regimes tested on “S” only).

### EFFECT OF DIET AND HIGH AND LOW MATERNAL EFFECT TREATMENTS ON ASSORTATIVE MATING, SEXUAL ISOLATION (PSI), SEXUAL SELECTION (PSS), AND TOTAL ISOLATION (PTI)

#### Effects of diet treatments at the start of selection (generation 5–7) on mating preferences

**Figure 5:**
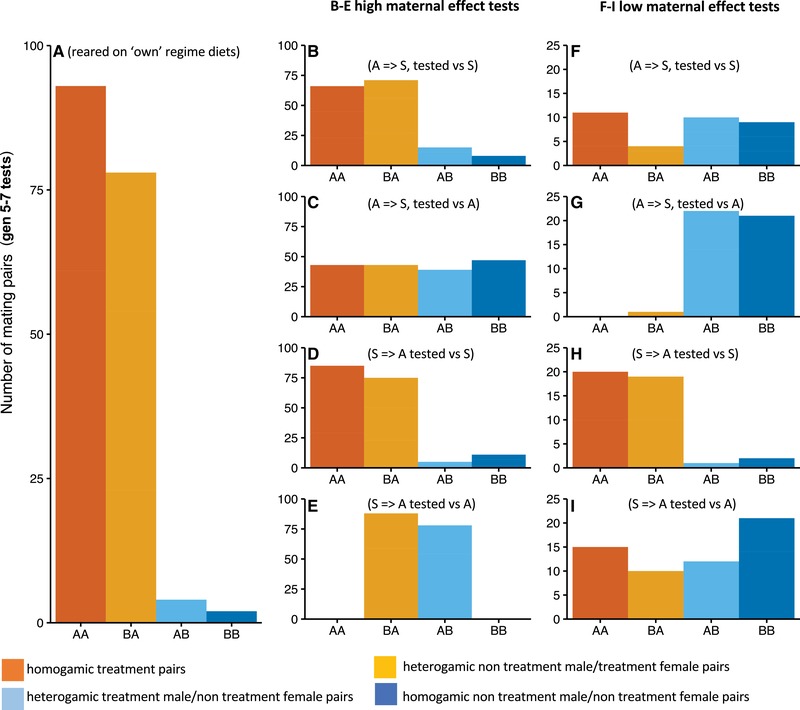
Quartet mating experiments between flies from the “A” and “S” dietary regimes, generation 5–7. Barplots representing the number of pairs formed in quartet mating tests between ASG (“A”) and Starch (“S”) dietary selection lines after five generations of selection. Panel A shows pairs formed when lines were tested following rearing own their own regime diets. Panels B–E represent flies from each diet regime, crossed to the opposing diet for one generation (high maternal effect), and tested against uncrossed individuals from each regime, as follows: panel B shows A regime flies crossed onto the S diet and tested against S regime flies (A = > S, tested vs S); panel C shows A regime flies crossed to the S diet tested against A regime flies (A = > S, tested vs A); panel D shows S flies crossed to A diet tested against S flies (S = > A tested vs S); panel E shows S flies crossed to A diet tested against A flies (S = > A tested vs A). Plots F–I represent flies from each diet regime, crossed to the opposing diet for two generations (low maternal effect), and tested against uncrossed individuals from each regime as follows: panel F is A regime flies crossed onto the S diet and tested against S regime flies (A = > S, tested vs S); panel G is A regime flies crossed to the S diet tested against A regime flies (A = > S, tested vs A); panel H is S flies crossed to A diet tested against S flies (S = > A tested vs S); panel I is S flies crossed to A diet tested against A flies (S = > A tested vs A). In each case, dark orange bars (AA) represent the number of homogamic treatment pairs; light orange bars (BA) heterogamic nontreatment male/treatment female pairs; light blue bars (AB) represent heterogamic treatment male/nontreatment female pairs; dark blue bars (BB) represent homogamic nontreatment male/nontreatment female pairs. For panel A, AA = homogamic ASG × ASG type matings, BB = homogamic Starch × Starch matings. Data from all three replicate lines are combined.

*Contribution of maternal effect variation*: There were marked effects of maternal effect variation on the pattern of mating (Fig. 5B–I). For example, the mating advantage of males reared or selected on “A” in the high maternal effect treatment disappeared under low maternal effects (panels B vs F, also evident in panel C vs G). Starch reared males lost matings to “A” competitors in the high maternal effect treatment (Fig. 5E), an effect that disappeared following two generations of rearing on “A” (low maternal effect, panel I). The results showed strong proximate effects of test diets and significant maternal effects. Overall, the findings are consistent with the predictions—the contribution of maternal carry over effects on male mating success was significantly diminished as dietary adaptation occurred under constant dietary regimes. Further details of specific initial responses are outlined below.

(i) *Mating tests on evolutionary regime diets*: At the start, all three replicates showed significant deviation from random preference when tested on own diets (PTI, *P < *0.001 in all cases, Table S4, Fig. 5A). “A” males were significantly more likely to mate with females from any source (PSS, *P < *0.001 in all cases, Table S4). PSI was not different from random mating (Table S4).
(ii) *Mating tests following rearing on opposing diets for one generation (high maternal effect)*: A/S flies with “S” competitors showed a significant deviation from random preference (PTI coefficient *P* < 0.001 in all cases, Table S4, Fig. 5B), which were mostly attributable to differences in sexual selection (PSS, *P* < 0.001 in all cases, Table S4). Despite being reared on the same testing diet, A/S males had a significant mating advantage over males whose parents were reared on Starch (S). By contrast A/S males placed with “A” competitors showed no significant deviation from random mating (PTI coefficient *P* > 0.05 in all cases; Table S4, Fig. 5C). This shows evidence for an initial influence of maternal effects on male mating success. In quartets containing S/A males tested against “S” males, there was also significant deviation from random mating (PTI, *P* < 0.001, Table S4) caused by a combination of sexual isolation and selection (PSS & PSI, *P* < 0.001). PSS accounted for the greater additive variance, with Starch males forming mating pairs more frequently (Fig. 5D). With quartets containing S/A males in tests against “A” males, there was a significant deviation from random preference (PTI, *P* < 0.001, Table S4) caused primarily by sexual isolation (PSI, *P* < 0.001, Table S4) with all pairs comprised of heterogamic matings (Fig. 5E). In all cases, PSS did not deviate significantly from 1 (Table S4).
(iii) *Mating tests following rearing on opposing diets for two generations (low maternal effect)*: A/S/S males (placed with “S” competitors) showed no deviation from random mating (PTI coefficient *P > *0.05; Table S4, Fig. 5F). Against “A” competitors, there was a highly significant deviation from random PTI (*P < *0.001 in all cases, Table S4). This effect was mainly due to sexual selection (PSS, *P < *0.001, Table S4). “A” males showed a strong mating advantage over A/S/S males and mated more frequently with females from any source (Fig. 5G). PSI was not significantly different from random for all mating pairs (Table S4). Quartets containing S/A/A and “S” flies showed significant deviation from random mating (PTI coefficient; *P *≤ 0.003 in all cases, Table S4, Fig. 5H) with the major contributor being sexual selection (PSS, *P < *0.001 in all cases, Table S4). Quartets containing S/A/A flies, with ASG males and females, deviated significantly from random mating in 2/3 replicates (PTI coefficients; Table S4, Fig. 5I).

#### Effects of diet treatments on generation 30 mating preferences

**Figure 6:**
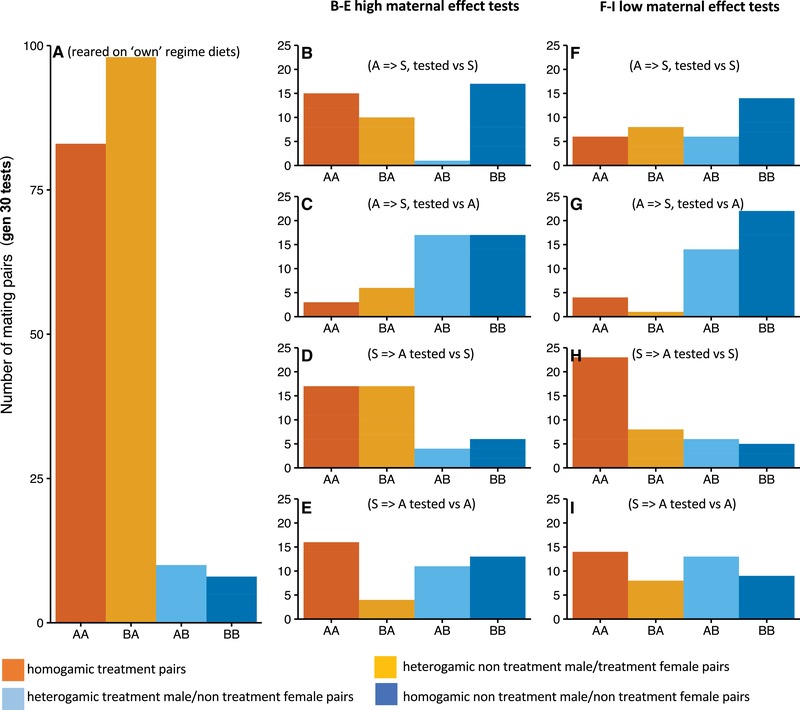
Quartet mating experiments between flies from the “A” and “S” dietary regimes, generation 30. Barplots representing the number of pairs formed in quartet mating tests between ASG (“A”) and Starch (“S”) dietary selection lines after 30 generations of selection. Panel A shows pairs formed when lines were tested following rearing own their own regime diets. Panels B–E represent flies from each diet regime, crossed to the opposing diet for one generation (high maternal effect), and tested against uncrossed individuals from each regime, as follows: panel B shows A regime flies crossed onto the S diet and tested against S regime flies (A = > S, tested vs S); panel C shows A regime flies crossed to the S diet tested against A regime flies (A = > S, tested vs A); panel D shows S flies crossed to A diet tested against S flies (S = > A tested vs S); panel E shows S flies crossed to A diet tested against A flies (S = > A tested vs A). Panels F–I represent flies from each regime, crossed to the opposing diet for two generations (low maternal effect), and tested against uncrossed individuals from each regime as follows: panel F is A regime flies crossed onto the S diet and tested against S regime flies (A = > S, tested vs S); panel G is A regime flies crossed to the S diet tested against A regime flies (A = > S, tested vs A); panel H is S flies crossed to A diet tested against S flies (S = > A tested vs S); panel I is S flies crossed to A diet tested against A flies (S = > A tested vs A). In each case, dark orange bars (AA) represent homogamic treatment male/treatment female pairs; light orange bars (BA) represent heterogamic nontreatment male/treatment female pairs; light blue bars (AB) represent heterogamic treatment male/nontreatment female pairs; dark blue bars (BB) represent homogamic nontreatment male/nontreatment female pairs. For panel A, AA = homogamic ASG × ASG type matings, BB = homogamic Starch × Starch matings. Data from all three replicate lines are combined.

*Contribution of maternal effect variation on male mating success at generation 30*: By generation 30, there was a marked lessening of the effects of maternal effect variation on male mating success. This was evident in the lack of differences between the pattern of matings observed in the high and low maternal effect treatments (Fig. 6 panels B–E versus F–I, respectively). Further specific details are given below.

(i) *Mating tests on own evolutionary regime diets*: Flies reared and tested on their own food showed similar patterns to those described above. There was a highly significant deviation from random mating in PTI (*P* < 0.001 in all cases, Table S4) again explained by strong differences in PSS (*P* < 0.001 in all cases, Table S4). There were no significant deviations from random in PSI. ASG males were significantly more likely to mate than were Starch males (Fig. 6A).
(ii) *Mating tests following rearing on opposing diets for one generation (high maternal effect)*: In contrast to the early generation tests, results from the mating tests of flies reared on the opposite diets were inconsistent and no general patterns emerged (Fig. 6B–E, Table S4).
(iii) *Mating tests following rearing on opposing diets for two generations (low maternal effect)*: Here, as in the high maternal effect treatments above, there was no consistent pattern (Table S4, Fig. 6F–I). Males maintained on “A” maintained a competitive advantage over “S” males. However, switching diets did not produce the earlier mating advantage, indicating that the lines may have undergone adaptation toward their respective diets, and that males did not gain a competitive advantage from switching to an “A” diet.

## Discussion

The results were broadly consistent with our predictions and showed that the contribution of maternal effects in responses to dietary selection evolved over time, being initially high and then diminishing significantly under the constant dietary selection regimes. There was also variation in the sensitivity of different traits to the magnitude of maternal effects, with developmental survival being relatively insensitive, developmental time demonstrating some evidence of maternal effects, and body mass and adult male mating success showing strong maternal effects. The influence of maternal effects on body size exhibited a significant interaction with the direction of diet switching tested, consistent with the idea that maternal effects confer most benefits when adult but not offspring dietary regimes are of good quality. Overall, as previously described (Leftwich et al. 2017), our results showed that the two larval rearing diets provided sufficiently distinct quantitative and qualitative dietary variation to drive the evolution of divergence, even in adult‐specific traits. The new contribution made by the current study is to show how maternal effects influenced this process. The results are discussed below in more detail, in terms of each of the original specific predictions.

### MATERNAL EFFECTS CONTRIBUTED TO DIETARY ADAPTATION

Maternal effects had strong influences on body size and male mating success at the start of selection, yet these effects had largely disappeared by generation 30—this suggests that the contribution of maternal effect variation had evolved during the study. This supports the idea that the sensitivity to maternal effects can change, presumably according to the ratio of fitness costs and benefits for mothers and offspring and this would be interesting to investigate further. We also cannot rule out that maternal effects remain, but are phenotypically silent as their effects are somehow masked by other facets of nutritional adaptation. It would be interesting to create additional evolutionary regimes that maintain dietary variation across generations, to test a corollary of our original prediction, that, should intergenerational dietary variation be maintained, maternal effects should also be maintained. In addition, further mechanistic insight into the nature of the full range of responses would be useful. For example, measures of the influence of egg size and content would be useful, as well as of the specific quantity and identity of maternal mRNAs loaded into eggs.

### MATERNAL EFFECTS DIMINISHED AS DIETARY CONDITIONS BECAME CONSTANT BETWEEN GENERATIONS

The decrease in strong maternal effects observed in some traits during nutritional selection is consistent with the prediction that selection on a fixed diet leads to a loss of benefits associated with maternal effect expression. For body mass in both sexes, the significant contribution of maternal effects at the start of selection was largely absent by generation 30. Male mating success followed a pattern that broadly echoed male body mass, with heavier males tending to mate more frequently. At the start of selection, the mating advantage of males reared or selected on “A” in the high maternal effect treatment disappeared under the low maternal effect treatment. “S” reared males also lost matings to “A” competitors in the high but not low maternal effect treatment. However, by generation 30, these strong maternal effects had again been lost. The results suggested that the short‐term proximate effects of diets, as well as the effects of maternal carry over on male mating success, were significantly diminished as dietary adaptation occurred. Overall, the results showed that the contribution of maternal effects to dietary responses in body mass and male mating success decreased over time, consistent with the original prediction.

### MATERNAL EFFECTS WERE TRAIT SPECIFIC

We observed that developmental survival was relatively insensitive to maternal effect variation both at the start and after selection. In contrast, development time, body mass, and male mating success were much more sensitive to high versus low maternal effects. These results are consistent with the original prediction that the influence of maternal effects should be trait specific. The precise reasons for trait specificity requires further investigation, but may reflect differences in trade‐offs between the chosen traits and other life‐history parameters.

### MATERNAL EFFECTS INTERACTED WITH HIGH AND LOW QUALITY FOOD

We observed effects consistent with maternal buffering effects associated with shifting from a good to a reduced calorie diet in the early generations. For example, there was an early influence of maternal effect variation on development time in “A” regime flies tested on “S.” Rearing on a Starch diet appeared to produce the fastest development time, but switching to the Starch diet from the “A” regime did not accelerate development, suggesting that maternal effects influenced how individuals responded to available carbohydrates. As for body size, this effect had disappeared by generation 30. At the start of experimental evolution, body mass was also significantly influenced by the strength of maternal effects, as evident in “A” regime flies tested on “S” diets (but not “S” on “A”). High maternal effect treatments retained heavier body mass characteristic of “A” regimes, while low maternal effect flies were significantly lighter. It is apparent from these results that the parental condition was effective at altering offspring condition. As noted above, by generation 30, maternal effects were not apparent and males and females had a higher body mass “on diet” in the later generations, while males in particular showed a significant drop in body mass for both “A” and “S” regimes under both high and low maternal effect treatments, indicating instead a reduction in fitness when reared off the diet to which they were adapted. The results are again consistent with this prediction and show that the contribution of maternal effects can be context dependent (e.g., for body mass apparent only in “A” regimes tested on “S”).

If maternal effects are adaptive, they can function to provision or prepare offspring in a manner that can help to maintain body size and male mating success, when moving onto diets with an altered nutrient profile. Conversely, the relevance of maternal effects to these traits became less marked as adaptation to the different diets occurred and as we observed a decay in the strength of the initial competitive advantage in male fitness associated with the “A” diet. Body mass and courtship intensity are strongly linked to male reproductive success in this and other species (Parker 1974; Partridge et al. 1987; Wiklund and Kaitala 1995; Pitnick and García‐González 2002; Leftwich et al. 2012). Our new results here show that there are different determinants underpinning the responses of both traits to variation in maternal effects. When diets are poor, maternal effects may be minimized because of the lack of resources available for mothers to place into the egg or embryo. Variation in calories between the two diets gives some indication into the likely selection pressures to which the founding population was challenged. The A diet had over twice the Kcal/L of S, and this was sufficient to lead to an early sexual advantage for the A males. The specific nutritional content of diets, rather than caloric content per se affects life‐history traits such as lifespan (Mair et al. 2005). Here, cornmeal in the A diet may have offered an additional source of carbohydrates, proteins, and other dietary nutrients (http://ndb.nal.usda.gov/). The competitive advantage seen by “A” males may have been mediated by this increase in diet content and complexity. However, it is also possible other nonnutritional factors associated with the diet, such as its consistency or other additives, could also have affected development or life history (Nash and Chapman 2014).

### MECHANISMS OF MATERNAL EFFECT VARIATION

The earliest stages of embryonic development begin under the control of mRNAs and proteins that have been deposited into the egg, usually maternal in origin (Preuss et al. 2012; Langley et al. 2014; Laver et al. 2015; Crofton et al. 2018). The composition of these maternal contributions therefore play a critical role in early development and can act as a mode of parental investment in offspring fitness, with mothers experiencing poor nutrition having more limited resources to devote to offspring fitness (Mousseau and Fox 1998). To confirm whether such effects were involved here, it would be useful to test in future studies to compare treatments for differences in egg size (Vijendravarma et al. 2010), as well as the composition of maternally deposited mRNAs and proteins in eggs (Tadros and Lipshitz 2009; Lockwood et al. 2017; Crofton et al. 2018).

### IMPORTANCE OF RESPONDING TO DIET

The medfly is a lekking species (Field et al. 2002) and reproductive success is strongly dependent on a male's competitive ability in a sexual context. Therefore, responses to environmental factors, such as diet, are likely to be key in determining a male's overall male reproductive success. Many studies have highlighted the importance of adult nutrition (mainly protein) on male mating success in the medfly (Blay and Yuval 1997; Kaspi and Yuval 2000; Shelly et al. 2002; Joachim‐Bravo et al. 2009; Costa et al. 2012). However, in this study the effects of larval, not adult diet were varied. Larval dietary nutrition is also essential for reproductive maturation and copulation success in medfly, an effect that may be mediated by body size (Kaspi et al. 2002; Costa et al. 2011). Our study provides evidence that maintaining medfly populations on different larval diets (effectively different hosts) can lead to variation in the expression of maternal effects on traits such as body size and male mating success.

Our study also confirms that the diet experienced by a holometabolous insect during development can directly influence developmental traits and mate choice, even when adult nutrition is controlled. Developmental conditions may be vital in programming either the pattern of resource allocation in adult life history, or in shaping the pathways through which condition is manifested (Cotton et al. 2004; Bonduriansky et al. 2015). Larval dietary nutrients have been shown to have significant effects on developmental life history in holometabolous insects (Nijhout 2003a), on resulting adult size (Nijhout 2003b; Edgar 2006), expression of adult sexual signals (Delcourt and Rundle 2011; Havens and Etges 2013) and secondary sexual characters (Bonduriansky et al. 2015). Our results link the importance of developmental nutrients to the expression of sexually selected traits in a nutritionally homogeneous adult environment (e.g., van Doorn et al. 2009).

It is likely that adaptation to the developmental dietary environment drives selection across a suite of traits, and here we have shown a significant contribution of maternal effects as part of this process. Whether the strength of this selection is sufficiently strong to cause divergence in the face of gene flow is not yet known outside of a laboratory setting. However, the potential for dietary (host) specialization within a global pest and extreme generalist has implications for the efficacy of programmes that seek to control medfly populations using mass‐reared laboratory strains. Our findings also offer opportunities for advancing our understanding of the role of developmental environment in the generation of divergence.

Associate Editor: S. Baird

Handling Editor: M. Servedio

## Supporting information


**Table S1A**. Egg to adult eclosion after 3‐5 generations of selection on ASG or Starch.
**Table S1B**. Egg to adult eclosion after 30 generations of selection on ASG or Starch.
**Table S1C**. Egg to pupation survival after 3‐5 generations of selection on ASG or Starch.
**Table S1D**. Egg to pupation survival after 30 generations of selection on ASG or Starch.
**Table S1E**. Pupation to adult eclosion after 3‐5 generations of selection on ASG or Starch.
**Table S1F**. Pupation to adult eclosion after 30 generations of selection on ASG or Starch.
**Table S2A**. Egg to adult development time after 3‐5 generations of selection on ASG or Starch.
**Table S2B**. Egg to adult development time after 30 generations of selection on ASG or Starch.
**Table S2C**. Egg to pupal development time after 3‐5 generations of selection on ASG or Starch.
**Table S2D**. Egg to pupal development time after 30 generations of selection on ASG or Starch.
**Table S2E**. Pupation to adult development time after 3‐5 generations of selection on ASG or Starch.
**Table S2F**. Pupation to adult development time after 30 generations of selection on ASG or Starch.
**Table S3A**. Male body mass at eclosion after 3‐5 generations of selection on ASG or Starch.
**Table S3B**. Female body mass at eclosion after 3‐5 generations of selection on ASG or Starch.
**Table S3C**. Male body mass at eclosion after 30 generations of selection on ASG or Starch.
**Table S3D**. Female body mass at eclosion after 30 generations of selection on ASG or Starch.
**Table S4**. Analysis of frequency, and PTI, PSS and PSI coefficients, for mating pairs.
