# Supplementary material for: Contribution of maternal effects to dietary selection in Mediterranean fruit flies
Source: Evolution. 2019 Jan 7;73(2):278–92. doi: 10.1111/evo.13664 (PMC6492002; doi:10.1111/evo.13664)
Supplement: Supplementary file 1 — Table S1A. Egg to adult eclosion after 3‐5 generations of selection on ASG or Starch. Table S1B. Egg to adult eclosion after 30 generations of selection on ASG or Starch. Table S1C. Egg to pupation survival after 3‐5 generations of selection on ASG or Starch. Table S1D. Egg to pupation survival after 30 generations of selection on ASG or Starch. Table S1E. Pupation to adult eclosion after 3‐5 generations of selection on ASG or Starch. Table S1F. Pupation to adult eclosion after 30 generations of selection on ASG or Starch. Table S2A. Egg to adult development time after 3‐5 generations of selection on ASG or Starch. Table S2B. Egg to adult development time after 30 generations of selection on ASG or Starch. Table S2C. Egg to pupal development time after 3‐5 generations of selection on ASG or Starch. Table S2D. Egg to pupal development time after 30 generations of selection on ASG or Starch. Table S2E. Pupation to adult development time after 3‐5 generations of selection on ASG or Starch. Table S2F. Pupation to adult development time after 30 generations of selection on ASG or Starch. Table S3A. Male body mass at eclosion after 3‐5 generations of selection on ASG or Starch. Table S3B. Female body mass at eclosion after 3‐5 generations of selection on ASG or Starch. Table S3C. Male body mass at eclosion after 30 generations of selection on ASG or Starch. Table S3D. Female body mass at eclosion after 30 generations of selection on ASG or Starch. Table S4. Analysis of frequency, and PTI, PSS and PSI coefficients, for mating pairs. [file EVO-73-278-s001.docx]

Contribution of maternal effects to dietary selection in Mediterranean fruit flies

**Philip T. Leftwich^1,2^*, William J. Nash^1,3^*, Lucy A. Friend,^1^ and Tracey Chapman^1,4^**

*Joint first authors

*^1^School of Biological Sciences, University of East Anglia, Norwich Research Park, Norwich, NR4 7TJ, UK.*

*^2^The Pirbright Institute, Woking, Surrey, GU24 0NF, UK.*

*^3^Evolutionary Genomics Group,* Earlham Institute, *Norwich Research Park, Norwich, NR4 7UZ, UK.*

*^4^Email: tracey.chapman@uea.ac.uk*

*Supporting Information*

#### Table S1A. Egg to adult eclosion after 3-5 generations of selection on ASG or Starch.

Generalised linear mixed model (after stepwise model reduction) of the proportion of medfly eggs that survived to adult eclosion, following ‘on diet’, high or low maternal effect treatment manipulations. Tests were done after 3-5 generations of selection on ASG and Starch regimes.

| Fixed effects | Estimate (SE) | z value | p-value |
| --- | --- | --- | --- |
| Intercept | 0.14(0.09) | 1.67 | 0.09 |
| Regime | -0.2(0.11) | -1.82 | 0.07 |
| Test | -0.34(0.1) | -3.8 | <0.001 |
| Regime * Test | 0.42(0.13) | 3.32 | <0.001 |
| Random effects | **Variance (SD)** |  |  |
| Obs | 0.14(0.37) |  |  |
| Line * Regime | 0.006(0.07) |  |  |
| Regime | 0 |  |  |

#### Table S1B. Egg to adult eclosion after 30 generations of selection on ASG or Starch.

Generalised linear mixed model (after stepwise model reduction) of the proportion of medfly eggs that survived to adult eclosion, following ‘on diet’, high or low maternal effect treatment manipulations. Tests were done after 30 generations of selection on ASG and Starch regimes.

| Fixed effects | Estimate (SE) | z value | p-value |
| --- | --- | --- | --- |
| Intercept | 0.69(0.09) | -7.77 | <0.001 |
| Regime | 1.01(0.12) | 8.01 | <0.001 |
| Random effects | **Variance (SD)** |  |  |
| Obs | <0.001(<0.001) |  |  |
| Line * Regime | <0.001(<0.001) |  |  |
| Regime | 0 |  |  |

#### Table S1C. Egg to pupation survival after 3-5 generations of selection on ASG or Starch.

Generalised linear mixed model (after stepwise model reduction) of the proportion of medfly eggs that survived to pupation, following ‘on diet’, high or low maternal effect treatment manipulations. Tests were done after 3-5 generations of selection on ASG and Starch regimes.

| Fixed effects | Estimate (SE) | z value | p-value |
| --- | --- | --- | --- |
| Intercept | 0.26(0.08) | 3.03 | 0.002 |
| Regime | -0.18(0.11) | -1.69 | 0.092 |
| Test | -0.33(0.09) | -3.57 | <0.001 |
| Regime * Test | 0.48(0.13) | 3.73 | <0.001 |
| Random effects | **Variance (SD)** |  |  |
| Obs | 0.15(0.38) |  |  |
| Line * Regime | 0.005(0.068) |  |  |
| Regime | 0 |  |  |

#### Table S1D. Egg to pupation survival after 30 generations of selection on ASG or Starch.

Generalised linear mixed model (after stepwise model reduction) of the proportion of medfly eggs that survived to pupation, following ‘on diet’, high or low maternal effect treatment manipulations. Tests were done after 30 generations of selection on ASG and Starch regimes.

| Fixed effects | Estimate (SE) | z value | p-value |
| --- | --- | --- | --- |
| Intercept | -0.57(0.14) | -3.95 | <0.001 |
| Regime | 1.2(0.17) | 7.17 | <0.001 |
| Parental | -0.32(0.22) | -1.45 | 0.15 |
| Test | -0.04(0.22) | -0.18 | 0.86 |
| Parental * Test | 0.63(0.29) | 2.16 | 0.031 |
| Random effects | **Variance (SD)** |  |  |
| Obs | 0.73(0.85) |  |  |
| Line * Regime | 0 |  |  |
| Regime | 0 |  |  |

#### Table S1E. Pupation to adult eclosion after 3-5 generations of selection on ASG or Starch.

Generalised linear mixed model (after stepwise model reduction) of the proportion of pupae that survived to adult eclosion, following ‘on diet’, high or low maternal effect treatment manipulations. Tests were done after 3-5 generations of selection on ASG and Starch regimes.

| Fixed effects | Estimate (SE) | z value | p-value |
| --- | --- | --- | --- |
| Intercept | 2.94(0.12) | 23 | <0.001 |
| Test | -0.38(0.09) | -4.01 | <0.001 |
| Random effects | **Variance (SD)** |  |  |
| Obs | 0.16(0.41) |  |  |
| Line * Regime | 0.04(0.21) |  |  |
| Regime | 0.009(0.1) |  |  |

#### Table S1F. Pupation to adult eclosion after 30 generations of selection on ASG or Starch.

Generalised linear mixed model (after stepwise model reduction) of the proportion of pupae that survived to adult eclosion, following ‘on diet’, high or low maternal effect treatment manipulations. Tests were done after 30 generations of selection on ASG and Starch regimes.

| Fixed effects | Estimate (SE) | z value | p-value |
| --- | --- | --- | --- |
| Intercept | 3.13(0.17) | 18.4 | <0.001 |
| Test | -1.13(0.14) | -7.87 | <0.001 |
| Random effects | **Variance (SD)** |  |  |
| Obs | 0.55(0.74) |  |  |
| Line * Regime | 0.11(0.33) |  |  |
| Regime | 0 |  |  |

#### Table S2A. Egg to adult development time after 3-5 generations of selection on ASG or Starch.

Generalised linear mixed model (after stepwise model reduction) of development time for medfly eggs to adult eclosion, following ‘on diet’, high or low maternal effect treatment manipulations. Tests were done after 3-5 generations of selection on ASG and Starch regimes.

| Fixed effects | Estimate (SE) | df | t value | p-value |
| --- | --- | --- | --- | --- |
| Intercept | 23.4(0.14) | 180 | 163.67 | <0.001 |
| Regime | -0.19(0.2) |  | -0.94 | 0.35 |
| Parental | -0.15(0.2) |  | -0.74 | 0.46 |
| Diet | -0.04(0.2) |  | -.22 | 0.83 |
| Regime * Diet | -1..03(0.29) |  | -3.59 | <0.001 |
| Parental * Diet | 0.65(0.28) |  | 2.26 | 0.02 |
| Random effects | **Variance (SD)** |  |  |  |
| Residual | 0.62(0.78) |  |  |  |
| Line * Regime | 0 |  |  |  |
| Regime | 0 |  |  |  |

#### Table S2B. Egg to adult development time after 30 generations of selection on ASG or Starch.

Generalised linear mixed model (after stepwise model reduction) of development time for medfly eggs to adult eclosion, following ‘on diet’, high or low maternal effect treatment manipulations. Tests were done after 30 generations of selection on ASG and Starch regimes.

| Fixed effects | Estimate (SE) | df | t value | p-value |
| --- | --- | --- | --- | --- |
| Intercept | 20.14(0.15) | 162 | 128.53 | <0.001 |
| Regime | -0.81(0.17) |  | -4.87 | <0.001 |
| Diet | 0.78(0.17) |  | 4.72 | <0.001 |
| Random effects | **Variance (SD)** |  |  |  |
| Residual | 0.99(0.99) |  |  |  |
| Line * Regime | 0 |  |  |  |
| Regime | 0 |  |  |  |

#### Table S2C. Egg to pupal development time after 3-5 generations of selection on ASG or Starch.

Generalised linear mixed model (after stepwise model reduction) of development time for medfly eggs to pupation, following ‘on diet’, high or low maternal effect treatment manipulations. Tests were done after 3-5 generations of selection on ASG and Starch regimes.

| Fixed effects | Estimate (SE) | df | t value | p-value |
| --- | --- | --- | --- | --- |
| Intercept | 15.46(0.17) | 180 | 90.56 | <0.001 |
| Regime | -0.1(0.21) |  | -0.46 | 0.64 |
| Diet | -0.46(0.21) |  | -2.19 | 0.03 |
| Regime * Diet | -1.31(0.3) |  | -4.45 | <0.001 |
| Random effects | **Variance (SD)** |  |  |  |
| Residual | 0.87(0.93) |  |  |  |
| Line * Regime | <0.001(<0.001) |  |  |  |
| Regime | 0 |  |  |  |

#### Table S2D. Egg to pupal development time after 30 generations of selection on ASG or Starch.

Generalised linear mixed model (after stepwise model reduction) of development time for medfly eggs to pupation, following ‘on diet’, high or low maternal effect treatment manipulations. Tests were done after 30 generations of selection on ASG and Starch regimes.

| Fixed effects | Estimate (SE) | df | t value | p-value |
| --- | --- | --- | --- | --- |
| Intercept | 11.72(0.12) | 162 | 94.69 | <0.001 |
| Diet | 0.69(0.13) |  | 5.38 | <0.001 |
| Random effects | **Variance (SD)** |  |  |  |
| Residual | 0.63(0.79) |  |  |  |
| Line * Regime | 0.03(0.17) |  |  |  |
| Regime | 0.005(0.069) |  |  |  |

#### Table S2E. Pupation to adult development time after 3-5 generations of selection on ASG or Starch.

Generalised linear mixed model (after stepwise model reduction) of development time for pupation to adult eclosion, following ‘on diet’, high or low maternal effect treatment manipulations. Tests were done after 3-5 generations of selection on ASG and Starch regimes.

| Fixed effects | Estimate (SE) | df | t value | p-value |
| --- | --- | --- | --- | --- |
| Intercept | 7.97(0.05) | 180 | 134.14 | <0.001 |
| Diet | 0.16(0.08) |  | 1.85 | 0.066 |
| Random effects | **Variance (SD)** |  |  |  |
| Residual | 0.31(0.5) |  |  |  |
| Line * Regime | <0.001(0.001) |  |  |  |
| Regime | 0 |  |  |  |

#### Table S2F. Pupation to adult development time after 30 generations of selection on ASG or Starch.

Generalised linear mixed model (after stepwise model reduction) of development time for pupae that survived to adult eclosion, following ‘on diet’, high or low maternal effect treatment manipulations. Tests were done after 30 generations of selection on ASG and Starch regimes.

| Fixed effects | Estimate (SE) | df | t value | p-value |
| --- | --- | --- | --- | --- |
| Intercept | 8.39(0.05) | 162 | 141.69 | <0.001 |
| Regime | -0.57(0.08) |  | -6.87 | <0.001 |
| Random effects | **Variance (SD)** |  |  |  |
| Residual | 0.28(0.53) |  |  |  |
| Line * Regime | 0 |  |  |  |
| Regime | <0.001(0.001) |  |  |  |

#### Table S3A. Male body mass at eclosion after 3-5 generations of selection on ASG or Starch.

Generalised linear mixed model (after stepwise model reduction) of male body mass at eclosion, following ‘on diet’, high or low maternal effect treatment manipulations. Tests were done after 3-5 generations of selection on ASG and Starch regimes.

| Fixed effects | Estimate (SE) | df | t value | p-value |
| --- | --- | --- | --- | --- |
| Intercept | 1.42(0.03) | 174 | 46.82 | <0.001 |
| Regime | -0.12(0.04) |  | -3 | 0.012 |
| Parental | -0.12(0.03) |  | -3.55 | <0.001 |
| Diet | -0.09(0.03) |  | -2.71 | 0.007 |
| Random effects | **Variance (SD)** |  |  |  |
| Residual | 0.034(0.19) |  |  |  |
| Line * Regime | <0.001(0.02) |  |  |  |
| Regime | 0 |  |  |  |

#### Table S3B. Female body mass at eclosion after 3-5 generations of selection on ASG or Starch.

Generalised linear mixed model (after stepwise model reduction) of female body mass at eclosion, following ‘on diet’, high or low maternal effect treatment manipulations. Tests were done after 3-5 generations of selection on ASG and Starch regimes.

| Fixed effects | Estimate (SE) | df | t value | p-value |
| --- | --- | --- | --- | --- |
| Intercept | 1.31(0.03) | 180 | 51.67 | <0.001 |
| Parental | 0.12(0.04) |  | 2.62 | 0.01 |
| Diet | 0.06(0.04) |  | 1.3 | 0.19 |
| Parental * Diet | -0.31(0.06) |  | -5.05 | <0.001 |
| Random effects | **Variance (SD)** |  |  |  |
| Residual | <0.001(<0.001) |  |  |  |
| Line * Regime | 0 |  |  |  |
| Regime | <0.001(0.001) |  |  |  |

#### Table S3C. Male body mass at eclosion after 30 generations of selection on ASG or Starch.

Generalised linear mixed model (after stepwise model reduction) of male body mass at eclosion, following ‘on diet’, high or low maternal effect treatment manipulations. Tests were done after 30 generations of selection on ASG and Starch regimes.

| Fixed effects | Estimate (SE) | df | t value | p-value |
| --- | --- | --- | --- | --- |
| Intercept | 1.72(0.03) | 162 | 49.75 | <0.001 |
| Regime | -0.37(0.05) |  | -7.5 | <0.001 |
| Parental | -0.04(0.04) |  | -0.91 | 0.36 |
| Diet | -0.55(0.04) |  | -11.3 | <0.001 |
| Regime * Diet | 0.67(0.07) |  | 9.65 | <0.001 |
| Parental * Diet | 0.21(0.07) |  | 3 | 0.003 |
| Random effects | **Variance (SD)** |  |  |  |
| Residual | <0.001(<0.001) |  |  |  |
| Line * Regime | <0.001(<0.001) |  |  |  |
| Regime | 0 |  |  |  |

#### Table S3D. Female body mass at eclosion after 30 generations of selection on ASG or Starch.

Generalised linear mixed model (after stepwise model reduction) of female body mass at eclosion, following ‘on diet’, high or low maternal effect treatment manipulations. Tests were done after 30 generations of selection on ASG and Starch regimes.

| Fixed effects | Estimate (SE) | df | t value | p-value |
| --- | --- | --- | --- | --- |
| Intercept | 1.82(0.02) | 156 | 73.5 | <0.001 |
| Regime | -0.14(0.03) |  | -4.4 | <0.001 |
| Diet | -0.09(0.03) |  | -3.4 | <0.001 |
| Regime * Diet | 0.19(0.04) |  | 4.81 | <0.001 |
| Random effects | **Variance (SD)** |  |  |  |
| Residual | 0.01(0.12) |  |  |  |
| Line * Regime | <0.001(0.02) |  |  |  |
| Regime | 0 |  |  |  |

#### Table S4. Analysis of frequency, and PTI, PSS and PSI coefficients, for mating pairs.

G statistics on mating pairs – values for PTI, PSS & PSI coefficients. The number of observed and total possible pairing was calculated for each replicate. These raw data were analysed with JMATING ver 1.0 (Carvajal-Rodriguez and Rolan-Alvarez 2006), to carry out non-parametric G tests to test for significant deviations from random matings. As the G test is additive it allows the significance of contributions to sexual isolation (GI testing PSI coefficient) and sexual selection (GT testing PSS coefficient) to total isolation (GT testing PTI coefficient) to be calculated.

| Mating quartets | Gen | variable | | value | df | p | sig |
| --- | --- | --- | --- | --- | --- | --- | --- |
| A1S1 | 5 | GI | 2.07 | | 1 | 0.15 |  |
| A1S1 | 5 | GS | 65.51 | | 2 | < 0.001 | *** |
| A1S1 | 5 | GT | 67.57 | | 3 | < 0.001 | *** |
| A2S2 | 5 | GI | 1.47 | | 1 | 0.23 |  |
| A2S2 | 5 | GS | 73.07 | | 2 | < 0.001 | *** |
| A2S2 | 5 | GT | 74.54 | | 3 | < 0.001 | *** |
| A3S3 | 5 | GI | 0.65 | | 1 | 0.42 |  |
| A3S3 | 5 | GS | 58.91 | | 2 | < 0.001 | *** |
| A3S3 | 5 | GT | 59.56 | | 3 | < 0.001 | *** |
| AS1A1 | 5 | GI | 0.18 | | 1 | 0.67 |  |
| AS1A1 | 5 | GS | 0.32 | | 2 | 0.85 |  |
| AS1A1 | 5 | GT | 0.49 | | 3 | 0.92 |  |
| AS1S1 | 5 | GI | 1.63 | | 1 | 0.2 |  |
| AS1S1 | 5 | GS | 36.57 | | 2 | < 0.001 | *** |
| AS1S1 | 5 | GT | 38.2 | | 3 | < 0.001 | *** |
| AS2A2 | 5 | GI | 2.73 | | 1 | 0.098 |  |
| AS2A2 | 5 | GS | 0.9 | | 2 | 0.64 |  |
| AS2A2 | 5 | GT | 3.63 | | 3 | 0.3 |  |
| AS2S2 | 5 | GI | 4.86 | | 1 | 0.027 | * |
| AS2S2 | 5 | GS | 37.75 | | 2 | < 0.001 | *** |
| AS2S2 | 5 | GT | 42.61 | | 3 | < 0.001 | *** |
| AS3A3 | 5 | GI | 0.14 | | 1 | 0.71 |  |
| AS3A3 | 5 | GS | 2.78 | | 2 | 0.25 |  |
| AS3A3 | 5 | GT | 2.92 | | 3 | 0.4 |  |
| AS3S3 | 5 | GI | 0.56 | | 1 | 0.45 |  |
| AS3S3 | 5 | GS | 17.9 | | 2 | < 0.001 | *** |
| AS3S3 | 5 | GT | 18.46 | | 3 | < 0.001 | *** |
| ASS1A1 | 5 | GI | 0 | | 1 | 1 |  |
| ASS1A1 | 5 | GS | 25.85 | | 2 | < 0.001 | *** |
| ASS1A1 | 5 | GT | 25.85 | | 3 | < 0.001 | *** |
| ASS1S1 | 5 | GI | 0 | | 1 | 1 |  |
| ASS1S1 | 5 | GS | 0.29 | | 2 | 0.87 |  |
| ASS1S1 | 5 | GT | 0.29 | | 3 | 0.96 |  |
| ASS2A2 | 5 | GI | 0 | | 1 | 1 |  |
| ASS2A2 | 5 | GS | 24.1 | | 2 | < 0.001 | *** |
| ASS2A2 | 5 | GT | 24.1 | | 3 | < 0.001 | *** |
| ASS2S2 | 5 | GI | 0.64 | | 1 | 0.42 |  |
| ASS2S2 | 5 | GS | 0.81 | | 2 | 0.67 |  |
| ASS2S2 | 5 | GT | 1.45 | | 3 | 0.69 |  |
| ASS3A3 | 5 | GI | 1.33 | | 1 | 0.25 |  |
| ASS3A3 | 5 | GS | 16.02 | | 2 | < 0.001 | *** |
| ASS3A3 | 5 | GT | 17.35 | | 3 | < 0.001 | *** |
| ASS3S3 | 5 | GI | 3.9 | | 1 | 0.048 | * |
| ASS3S3 | 5 | GS | 2.05 | | 2 | 0.36 |  |
| ASS3S3 | 5 | GT | 5.95 | | 3 | 0.11 |  |
| SA1A1 | 5 | GI | 78.16 | | 1 | < 0.001 | *** |
| SA1A1 | 5 | GS | 1.72 | | 2 | 0.42 |  |
| SA1A1 | 5 | GT | 79.88 | | 3 | < 0.001 | *** |
| SA1S1 | 5 | GI | 8.91 | | 1 | 0.003 | ** |
| SA1S1 | 5 | GS | 17.75 | | 2 | < 0.001 | *** |
| SA1S1 | 5 | GT | 26.66 | | 3 | < 0.001 | *** |
| SA2A2 | 5 | GI | 77.56 | | 1 | < 0.001 | *** |
| SA2A2 | 5 | GS | 0.14 | | 2 | 0.93 |  |
| SA2A2 | 5 | GT | 77.7 | | 3 | < 0.001 | *** |
| SA2S2 | 5 | GI | 18 | | 1 | < 0.001 | *** |
| SA2S2 | 5 | GS | 15.3 | | 2 | < 0.001 | *** |
| SA2S2 | 5 | GT | 33.3 | | 3 | < 0.001 | *** |
| SA3A3 | 5 | GI | 73 | | 1 | < 0.001 | *** |
| SA3A3 | 5 | GS | 0.94 | | 2 | 0.63 |  |
| SA3A3 | 5 | GT | 73.95 | | 3 | < 0.001 | *** |
| SA3S3 | 5 | GI | 2.21 | | 1 | 0.14 |  |
| SA3S3 | 5 | GS | 47.56 | | 2 | < 0.001 | *** |
| SA3S3 | 5 | GT | 49.77 | | 3 | < 0.001 | *** |
| SAA1A1 | 5 | GI | 0.84 | | 1 | 0.36 |  |
| SAA1A1 | 5 | GS | 0.81 | | 2 | 0.67 |  |
| SAA1A1 | 5 | GT | 1.65 | | 3 | 0.65 |  |
| SAA1S1 | 5 | GI | 0 | | 1 | 1 |  |
| SAA1S1 | 5 | GS | 27.73 | | 2 | < 0.001 | *** |
| SAA1S1 | 5 | GT | 27.73 | | 3 | < 0.001 | *** |
| SAA2A2 | 5 | GI | 1.28 | | 1 | 0.26 |  |
| SAA2A2 | 5 | GS | 0.53 | | 2 | 0.77 |  |
| SAA2A2 | 5 | GT | 1.81 | | 3 | 0.61 |  |
| SAA2S2 | 5 | GI | 1.44 | | 1 | 0.23 |  |
| SAA2S2 | 5 | GS | 19.79 | | 2 | < 0.001 | *** |
| SAA2S2 | 5 | GT | 21.22 | | 3 | < 0.001 | *** |
| SAA3A3 | 5 | GI | 1.28 | | 1 | 0.26 |  |
| SAA3A3 | 5 | GS | 0.53 | | 2 | 0.77 |  |
| SAA3A3 | 5 | GT | 1.81 | | 3 | 0.61 |  |
| SAA3S3 | 5 | GI | 0.06 | | 1 | 0.81 |  |
| SAA3S3 | 5 | GS | 14.03 | | 2 | < 0.001 | *** |
| SAA3S3 | 5 | GT | 14.08 | | 3 | 0.003 | ** |
| A1S1 | 30 | GI | 0.01 | | 1 | 0.92 |  |
| A1S1 | 30 | GS | 59.13 | | 2 | < 0.001 | *** |
| A1S1 | 30 | GT | 59.13 | | 3 | < 0.001 | *** |
| A2S2 | 30 | GI | 0.15 | | 1 | 0.7 |  |
| A2S2 | 30 | GS | 67.57 | | 2 | < 0.001 | *** |
| A2S2 | 30 | GT | 67.73 | | 3 | < 0.001 | *** |
| A3S3 | 30 | GI | 0.16 | | 1 | 0.69 |  |
| A3S3 | 30 | GS | 36.31 | | 2 | < 0.001 | *** |
| A3S3 | 30 | GT | 36.47 | | 3 | < 0.001 | *** |
| AS1A1 | 30 | GI | 0.61 | | 1 | 0.43 |  |
| AS1A1 | 30 | GS | 5.85 | | 2 | 0.054 |  |
| AS1A1 | 30 | GT | 6.46 | | 3 | 0.091 |  |
| AS1S1 | 30 | GI | 9.11 | | 1 | 0.003 | ** |
| AS1S1 | 30 | GS | 2.94 | | 2 | 0.23 |  |
| AS1S1 | 30 | GT | 12.06 | | 3 | 0.007 | ** |
| AS2A2 | 30 | GI | 0.07 | | 1 | 0.79 |  |
| AS2A2 | 30 | GS | 6.39 | | 2 | 0.041 | * |
| AS2A2 | 30 | GT | 6.46 | | 3 | 0.091 |  |
| AS2S2 | 30 | GI | 0.63 | | 1 | 0.43 |  |
| AS2S2 | 30 | GS | 3.4 | | 2 | 0.18 |  |
| AS2S2 | 30 | GT | 4.03 | | 3 | 0.26 |  |
| AS3A3 | 30 | GI | 0 | | 1 | 1 |  |
| AS3A3 | 30 | GS | 22 | | 2 | < 0.001 | *** |
| AS3A3 | 30 | GT | 22 | | 3 | < 0.001 | *** |
| AS3S3 | 30 | GI | 7.82 | | 1 | 0.005 | ** |
| AS3S3 | 30 | GS | 6.02 | | 2 | 0.049 | * |
| AS3S3 | 30 | GT | 13.84 | | 3 | 0.003 | ** |
| ASS1A1 | 30 | GI | 0 | | 1 | 1 |  |
| ASS1A1 | 30 | GS | 24.19 | | 2 | < 0.001 | *** |
| ASS1A1 | 30 | GT | 24.19 | | 3 | < 0.001 | *** |
| ASS1S1 | 30 | GI | 1.28 | | 1 | 0.26 |  |
| ASS1S1 | 30 | GS | 8.96 | | 2 | 0.011 | * |
| ASS1S1 | 30 | GT | 10.24 | | 3 | 0.017 | * |
| ASS2A2 | 30 | GI | 2.26 | | 1 | 0.13 |  |
| ASS2A2 | 30 | GS | 1.77 | | 2 | 0.41 |  |
| ASS2A2 | 30 | GT | 4.03 | | 3 | 0.26 |  |
| ASS2S2 | 30 | GI | 1.6 | | 1 | 0.21 |  |
| ASS2S2 | 30 | GS | 3.19 | | 2 | 0.2 |  |
| ASS2S2 | 30 | GT | 4.79 | | 3 | 0.19 |  |
| ASS3A3 | 30 | GI | 0 | | 1 | 1 |  |
| ASS3A3 | 30 | GS | 15.34 | | 2 | < 0.001 | *** |
| ASS3A3 | 30 | GT | 15.34 | | 3 | 0.002 | ** |
| ASS3S3 | 30 | GI | 2.06 | | 1 | 0.15 |  |
| ASS3S3 | 30 | GS | 5.65 | | 2 | 0.059 |  |
| ASS3S3 | 30 | GT | 7.7 | | 3 | 0.053 |  |
| SA1A1 | 30 | GI | 0.42 | | 1 | 0.52 |  |
| SA1A1 | 30 | GS | 1.21 | | 2 | 0.55 |  |
| SA1A1 | 30 | GT | 1.62 | | 3 | 0.65 |  |
| SA1S1 | 30 | GI | 2.25 | | 1 | 0.13 |  |
| SA1S1 | 30 | GS | 9.62 | | 2 | 0.008 | ** |
| SA1S1 | 30 | GT | 11.86 | | 3 | 0.008 | ** |
| SA2A2 | 30 | GI | 0.2 | | 1 | 0.65 |  |
| SA2A2 | 30 | GS | 9.08 | | 2 | 0.011 | * |
| SA2A2 | 30 | GT | 9.28 | | 3 | 0.026 | * |
| SA2S2 | 30 | GI | 0.03 | | 1 | 0.86 |  |
| SA2S2 | 30 | GS | 1.45 | | 2 | 0.48 |  |
| SA2S2 | 30 | GT | 1.47 | | 3 | 0.69 |  |
| SA3A3 | 30 | GI | 4.14 | | 1 | 0.042 | * |
| SA3A3 | 30 | GS | 9.62 | | 2 | 0.008 | ** |
| SA3A3 | 30 | GT | 13.76 | | 3 | 0.003 | ** |
| SA3S3 | 30 | GI | 2.25 | | 1 | 0.13 |  |
| SA3S3 | 30 | GS | 9.62 | | 2 | 0.008 | ** |
| SA3S3 | 30 | GT | 11.86 | | 3 | 0.008 | ** |
| SAA1A1 | 30 | GI | 1.77 | | 1 | 0.18 |  |
| SAA1A1 | 30 | GS | 0.13 | | 2 | 0.94 |  |
| SAA1A1 | 30 | GT | 1.9 | | 3 | 0.59 |  |
| SAA1S1 | 30 | GI | 0.93 | | 1 | 0.33 |  |
| SAA1S1 | 30 | GS | 1.45 | | 2 | 0.48 |  |
| SAA1S1 | 30 | GT | 2.38 | | 3 | 0.5 |  |
| SAA2A2 | 30 | GI | 2.95 | | 1 | 0.086 |  |
| SAA2A2 | 30 | GS | 1.16 | | 2 | 0.56 |  |
| SAA2A2 | 30 | GT | 4.11 | | 3 | 0.25 |  |
| SAA2S2 | 30 | GI | 0.77 | | 1 | 0.38 |  |
| SAA2S2 | 30 | GS | 12.94 | | 2 | 0.002 | ** |
| SAA2S2 | 30 | GT | 13.72 | | 3 | 0.003 | ** |
| SAA3A3 | 30 | GI | 6.69 | | 1 | 0.01 | ** |
| SAA3A3 | 30 | GS | 6.39 | | 2 | 0.041 | * |
| SAA3A3 | 30 | GT | 13.08 | | 3 | 0.004 | ** |
| SAA3S3 | 30 | GI | 1.42 | | 1 | 0.23 |  |
| SAA3S3 | 30 | GS | 6.79 | | 2 | 0.034 | * |
| SAA3S3 | 30 | GT | 8.22 | | 3 | 0.042 | * |

**LITERATURE CITED**

Carvajal-Rodriguez, A., and E. Rolan-Alvarez. 2006. JMATING: a software for the analysis of sexual selection and sexual isolation effects from mating frequency data. BMC Evol. Biol. 6:40.
